# Effects of different operating parameters on hydrogen production by *Parageobacillus thermoglucosidasius* DSM 6285

**DOI:** 10.1186/s13568-019-0931-1

**Published:** 2019-12-23

**Authors:** Teresa Mohr, Habibu Aliyu, Lars Biebinger, Roman Gödert, Alexander Hornberger, Don Cowan, Pieter de Maayer, Anke Neumann

**Affiliations:** 1 Section II: Technical Biology, Institute of Process Engineering in Life Science, Karlsruhe Institute of Technology, 76131 Karlsruhe, Germany; 2 Department of Biochemistry, Genetics and Microbiology, Centre for Microbial Ecology and Genomics, University of Pretoria, Hatfield, Pretoria, 0028 South Africa; 3 School of Molecular & Cell Biology, Faculty of Science, University of the Witwatersrand, Johannesburg, South Africa

**Keywords:** *Parageobacillus thermoglucosidasius*, Water–gas shift reaction, Biohydrogen, Process optimization, CO-dehydrogenase

## Abstract

Hydrogen gas represents a promising alternative energy source to dwindling fossil fuel reserves, as it carries the highest energy per unit mass and its combustion results in the release of water vapour as only byproduct. The facultatively anaerobic thermophile *Parageobacillus thermoglucosidasius* is able to produce hydrogen via the water–gas shift reaction catalyzed by a carbon monoxide dehydrogenase–hydrogenase enzyme complex. Here we have evaluated the effects of several operating parameters on hydrogen production, including different growth temperatures, pre-culture ages and inoculum sizes, as well as different pHs and concentrations of nickel and iron in the fermentation medium. All of the tested parameters were observed to have a substantive effect on both hydrogen yield and (specific) production rates. A final experiment incorporating the best scenario for each tested parameter showed a marked increase in the H_2_ production rate compared to each individual parameter. The optimised parameters serve as a strong basis for improved hydrogen production with a view of commercialisation of this process.

## Introduction

Hydrogen (H_2_) gas is a critical component of diverse industrial applications including the synthesis of ammonia, methanol production and petroleum processing (Ramachandran and Menon 1998). Furthermore, H_2_ is an efficient energy carrier as, compared to fossil fuel, it has higher energy per unit mass and its combustion produces zero toxic emissions (CO_2_, SO_2_ and NOx). Consequently, H_2_ has been projected as a formidable energy alternative to dwindling fossil fuel reserves and has become an important component of global energy dynamics (Nikolaidis and Poullikkas 2017). Currently, large-scale H_2_ production is performed via several mechanisms, including natural gas reformation, where carbon atoms from methane separate when exposed to steam and heat, resulting in the release of H_2_ and carbon monoxide (CO) (Sørensen and Spazzafumo 2011). Other commonly applied approaches include gasification of coal (to H_2_ and CO) and electrolysis of water (to H_2_ and O_2_). However, these methods are costly, often use fossil fuels and have harmful environmental effects (Nikolaidis and Poullikkas 2017). As such, several biological strategies for hydrogen production have been explored including photofermentation by photosynthetic bacteria, bio-photolysis of water by algae and dark fermentation of organic substances by anaerobic microorganisms (Sokolova et al. 2009). Recently, there has been increased interest in microorganisms that produce H_2_ via the water–gas shift reaction (WGS): CO + H_2_O → CO_2_ + H_2_ (Diender et al. 2015; Mohr et al. 2018a). The WGS reaction couples the oxidation of CO with the splitting of a water molecule to yield CO_2_ and H_2_ gas (Tirado-Acevedo et al. 2010). This is particularly pertinent as these microorganisms can use syngas, a natural product of steam reformation of natural gas and gasification of coal and municipal waste, which primarily consists of CO, CO_2_ and H_2_ (Rostrup-Nielsen 1993). The thermophilic bacterium *P. thermoglucosidasius* DSM 6285 produces H_2_ via the WGS reaction using a carbon monoxide dehydrogenase—NiFe group 4a hydrogenase complex (Mohr et al. 2018b). In contrast to anaerobic organisms, *Parageobacillus thermoglucosidasius* is a facultative anaerobe which tolerates high concentrations of both CO and O_2_, first growing aerobically until O_2_ is depleted followed by the anaerobic WGS reaction. However, a lag phase was observed between O_2_ depletion and commencement of H_2_ production (Mohr et al. 2018a, b). In the current study the effects of different process parameters on H_2_ production were investigated in batch experiments. The optimized parameters will form the basis for further development of up-scale biological hydrogen production with *P. thermoglucosidasius*.

## Materials and methods

### Microorganism and medium

*Parageobacillus thermoglucosidasius* DSM 6285 was obtained from the Deutsche Sammlung von Mikroorganismen und Zellkulturen (DSMZ, Braunschweig, Germany) and stored at − 80 °C in glycerol (80%) stocks. The cultivation of *P. thermoglucosidasius* DSM 6285 was performed in 50 mLB (modified Luria–Bertani) medium (Zeigler 2001). This medium contains tryptone (10 g/L), yeast extract (5 g/L), NaCl (5 g/L), 1.25 mL/L NaOH (10 g/L) and 1 mL/L of each of the filter-sterilized stock solutions 1.05 M nitrilotriacetic acid, 0.59 M MgSO_4_·7H_2_O, 0.91 M CaCl_2_·2H_2_O and 0.04 M FeSO_4_·7H_2_O.

### Inoculum preparation

A two-step pre-culture approach was adopted for this study. In the first pre-culture, 20 mL mLB medium were inoculated with 20 µL of glycerol stock and cultivated for 24 h. A second pre-culture was inoculated from the first to an initial absorbance (OD_600_) of 0.1. All pre-cultures were cultivated aerobically in 100 mL shake flasks containing 20 mL mLB medium at 60 °C and 120 rpm (Infors Thermotron, Infors AG, Bottmingen, Switzerland). After 12 h, an appropriate amount of the 2nd pre-culture (2%, 10% and 20% v/v) was added to 250 mL stoppered serum bottles (containing 50 mL mLB medium total) in an appropriate initial gas atmosphere ratio (36:64, 50:50 and 75:25) of CO and air at 1 bar atmospheric pressure (at 25 °C). Air was required during all set-ups to ensure biomass production prior to the anaerobic H_2_ production. The cultivations were performed in triplicate for a duration of 82 h.

### Experimental set up

The effects of different operational parameters on *P. thermoglucosidasius* H_2_ production were investigated as per Table 1. To examine the effects of temperature and pH on growth and hydrogen production, the cultures were maintained at 50 °C, 55 °C and 60 °C. The pH was adjusted to 5.5, 7.0 and 8.5 using either NaOH (1 M) or HCl (1 M). Both the CODH and group 4a hydrogenase in *P. thermoglucosidasius* are comprised of a Ni–Fe metallocenter (Mohr et al. 2018a). To determine the effects of higher iron (Fe^2+^) concentrations on hydrogenogenesis, double the amount of FeSO_4_·7H_2_O (0.08 mM) normally included in mLB medium (0.04 mM; Mohr et al. 2018a) was added in one experimental set-up. As the mLB medium does not include the addition of nickel (Ni^2+^), one set-up was prepared containing 0.3 mM NiSO_4_·6H_2_O. The results were compared to those obtained by growing *P. thermoglucosidasius* DSM 6285 in mLB containing only 0.04 mM FeSO_4_·7H_2_O and no exogenous nickel. The effects of different initial gas compositions on H_2_ production were also evaluated using 36:64, 50:50 and 75:25 CO:air ratios. The influence of incubation time and volume of the inoculum were studied by varying the incubation times of the 2nd pre-culture from 4 h, 12 h to 24 h and by using inoculum volumes of the 2nd pre-culture of 2%, 10% and 20% of the final volume (50 mL).

**Table 1:**
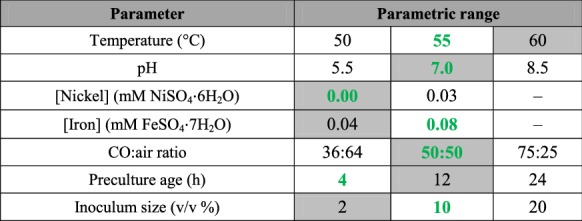
Parameters evaluated in the study

Values shaded in grey represent those used in previous experiments (Mohr et al., 2018a, b). Values in bold and green represent those which gave highest H_2_ productivity and/or H_2_ yield)

To investigate the combination of the parameters which resulted in a superior hydrogen production, a further experiment was conducted. Here, one condition for each parameter was chosen based on the maximum production rate and highest obtained yield: 55 °C, pH 7.0 (initial), addition of FeSO_4_·7H_2_O (0.08 mM), 75:25 CO:air ratios (initial gas atmosphere), 4 h incubation time of the 2nd pre-culture, 10% inoculum size. To validate whether the tested parameters have a positive effect on the H_2_ production, the experimental set up as in Mohr et al. (2018b) was used as a control: 60 °C growth temperature, pH 7.0, 0.04 mM FeSO_4_·7H_2_O, 50:50 CO:air ratios (initial gas atmosphere), 12 h incubation time of the 2nd pre-culture and 2% inoculum volume.

### Analytical methods

To determine growth, 1 mL of culture was removed from the bottles through the stoppers using a sterile needle and syringe and absorbance (OD_600_) was measured using an Ultrospec 1100 pro spectrophotometer (Amersham Biosciences, USA). The medium pH was determined from the same sample using a Profilab pH 597 pH meter (Xylem Analytics Germany Sales GmbH & Co. KG, WTW, Germany). OD_600_ of 1 equates to 0.3472 g/L bio dry weight. To measure the gas compositions at each time point, a 3 mL gas sample was taken from the head-space of the bottle and injected to a 300 Micro GC gas analyzer (Inficon, Bad Ragaz, Switzerland), fitted with the columns Molsieve and PLOT Q. The column temperature was maintained at 80 °C for a duration of 180 s. Pressure was measured using a manometer (GDH 14 AN, Greisinger electronic, Regenstauf, Germany) prior to and after each sample was extracted from the bottles.

### Data analysis

Gas compositions were calculated on the basis of the ideal gas law as previously described (Mohr et al. 2018a). In order to compare the results of the different process parameters, hydrogen production rates between different sampling time points were calculated as per Eq. 1:

1 $$ {\text{Production}}\;{\text{rate}} = \frac{{\Delta  m_{\text{hydrogen}} \left[ {\text{mmol}} \right]}}{{\Delta  time \left[ {\text{h}} \right]}}. $$

The specific production rate was calculated as per Eq. 2:

2 $$ {\text{Specific}}\;{\text{production}}\;{\text{rate}} = \frac{{\Delta m_{\text{hydrogen}} \left[ {\text{mmol}} \right]}}{{\Delta  time \left[ {\text{h}} \right]*OD_{600} }}. $$

The overall H_2_ yield for each of the experiments was calculated as a function of CO consumption (Eq. 3). This was done for the hydrogenogenic phase from the first time point where H_2_ was detected (24 h post-inoculation) until the CO was consumed in most experimental set-ups (72 h post-inoculation).

3 $$ {\text{Yield}} = \frac{{\Delta  {\text{H}}2\left[ {\text{mmol}} \right]}}{{\Delta  {\text{CO}} \left[ {\text{mmol}} \right]}}. $$

## Results

### Effect of initial gas composition on H_2_ productivity

**Fig. 1:**
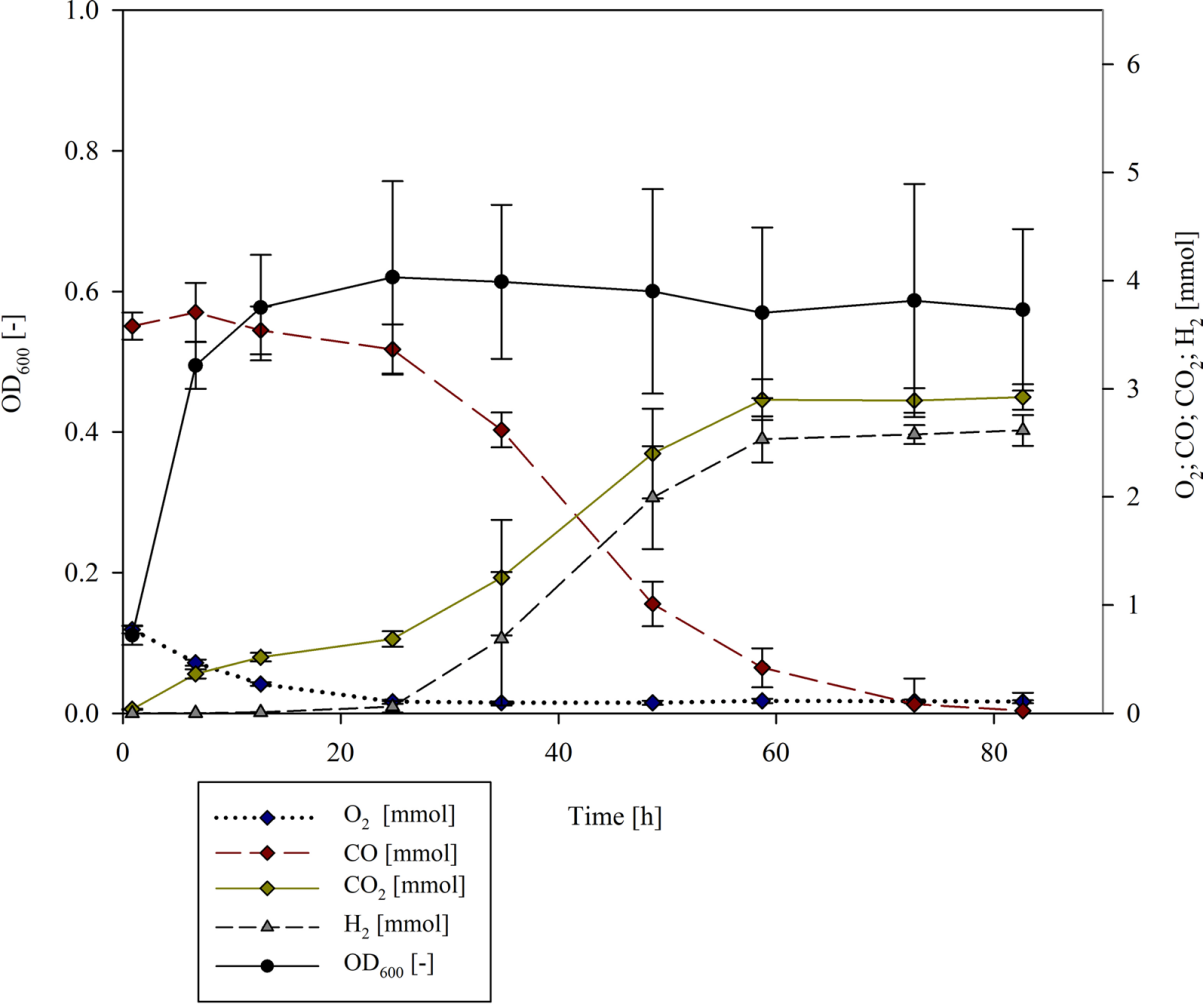
Growth curve and gas composition during the cultivation of *P. thermoglucosidasius* DSM 6285 in the control set up (60 °C, pH 7.0, addition of 0.04 mM FeSO_4_·7H_2_O, 50:50 initial gas atmosphere CO:air ratios, 12 h incubation time of the 2nd pre-culture, 2% inoculum size)

**Fig. 2:**
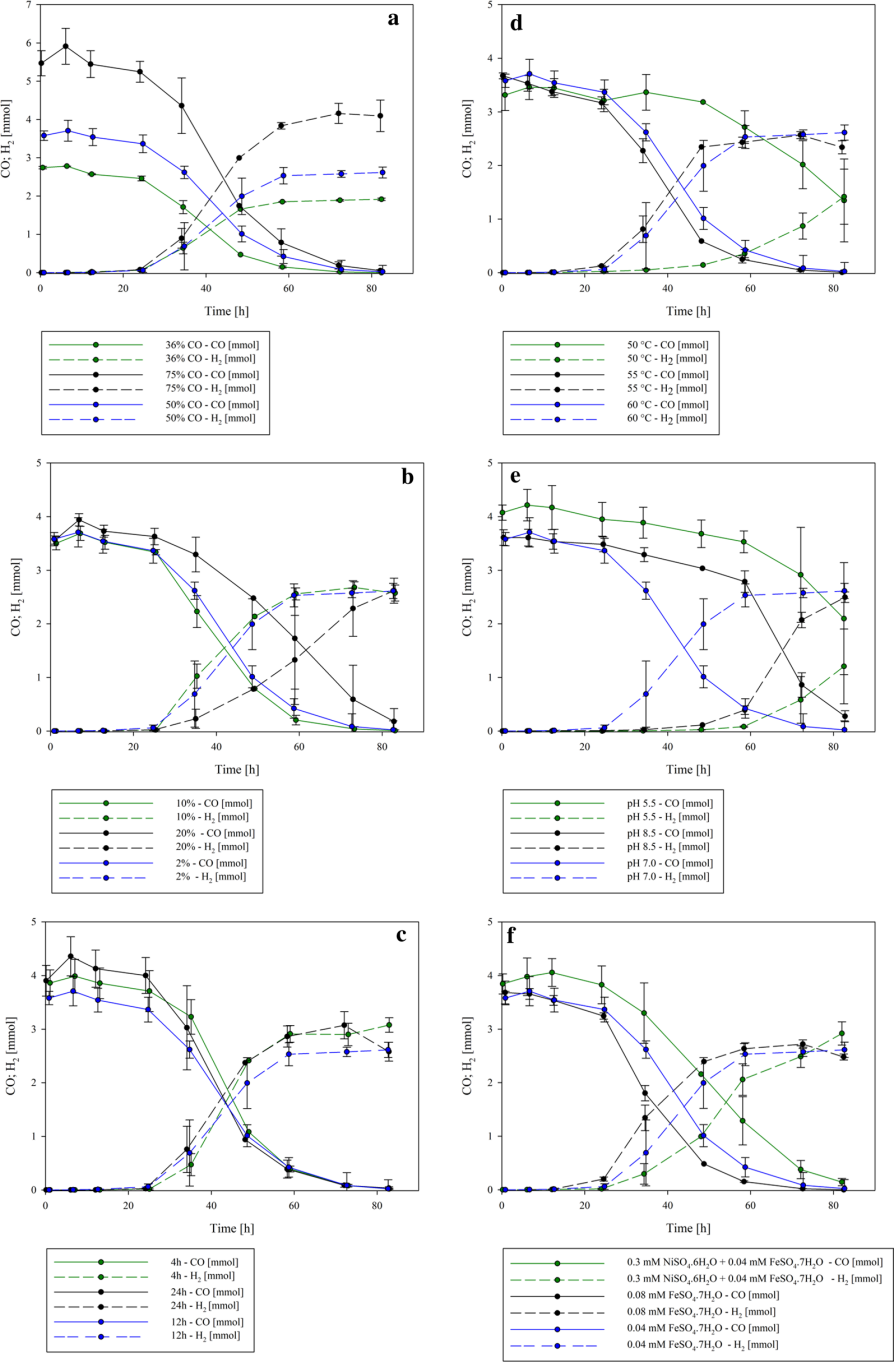
Effects of several operating parameters on CO consumption and H_2_ production during the cultivation of *P. thermoglucosidasius*. **a** Initial gas composition, **b** inoculum size, **c** age of 2nd pre-culture, **d** temperature, **e** initial pH and **f** addition of FeSO_4_·7H_2_O and NiSO_4_·6H_2_O

To evaluate the effect of the initial gas composition, H_2_ production with three distinct CO:air ratios (36:64, 50:50 and 75:25) was determined. Spectrophotometric analysis of the biomass showed that, while *P. thermoglucosidasius* DSM 6285 grown in the 36:64 and 50:50 CO:air gas ratios grew to a maximum absorbance of 0.744 ± 0.103 (after 24 h) and 0.620 ± 0.137 (after 24 h), respectively, it grew substantially less and at a slower rate with a 75:25 CO:air ratio, with a maximum absorbance of 0.476 ± 0.028 after 72 h (Fig. 1, Additional file 1). This suggests that the lower O_2_ concentration affected effective biomass formation in the initial aerobic phase. O_2_ reached its minimum for all tested gas compositions after ~ 24 h, while H_2_ was initially detected at approximately the same time. CO was completely consumed at the end of the cultivations in all instances (Fig. 2a). Maximum H_2_ production rates were observed between the 34 h and 48 h sample points in all cases. The highest values were observed for *P. thermoglucosidasius* exposed to the 75:25% atmosphere, with 0.138 ± 0.009 mmol/h H_2_ produced in this time frame. By contrast, substantially lower maximum production rates were observed with 36% and 50% CO in the initial gas atmosphere (0.073 ± 0.006 mmol/h and 0.094 ± 0.016 mmol/h, respectively) (Table 2). With an increasing initial CO concentration, the specific productivity increases from 0.104 ± 0.016 (36% CO), 0.163 ± 0.054 (50% CO) to 0.351 ± 0.038 (75% CO). The overall H_2_ yield during the hydrogenogenic phase was also higher with the 75% CO concentration (0.807 ± 0.022 mmol H_2_/mmol CO) than when 50% and 36% CO were present in the bottles (5.45 and 7.31% higher, respectively) (Table 2).

**Table 2:** Hydrogen production rate (mmol/h), specific production rate (mmol/h/OD_600_), yield (H_2_ mmol/CO mmol) and growth rates for the individual and combined parameters

Parameter	Production rate		Specific production rate		Yield^a^ (H_2_ mmol/CO mmol)	Aerobic growth phase	
	Maximum (mmol/h)	Time (h)	Maximum (mmol/h * OD_600_)	Time (h)		∆OD_600_ (t12 − t0)	µ (1/h)
Control: 60 °C; pH 7.0; 0.04 mM Fe^2+^; no Ni^2+^; 50% CO; 12 h pre-culture; 2% inoculum	0.094 ± 0.016	35–48	0.163 ± 0.054	35–48	0.763 ± 0.026	0.467 ± 0.073	0.14
50 °C	0.055 ± 0.027	73–82	0.144 ± 0.06	73–82	0.698 ± 0.068	0.647 ± 0.044	0.163
55 °C	0.098 ± 0.006	34–48	0.124 ± 0.001	34–48	0.783 ± 0.014	0.716 ± 0.059	0.172
pH 5.5	0.06 ± 0.025	72–82	0.358 ± 0.103	72–82	0.606 ± 0.168	0.127 ± 0.035	0.128
pH 8.5	0.122 ± 0.005	59–72	0.294 ± 0.028	59–72	0.786 ± 0.018	0.348 ± 0.006	0.127
0.3 mM NiSO_4_·6H_2_O + 0.04 mM FeSO4·7H_2_O	0.078 ± 0.015	48–58	0.192 ± 0.035	48–58	0.716 ± 0.032	0.36 ± 0.056	0.122
0.08 mM FeSO_4_·7H_2_O	0.115 ± 0.020	25–35	0.24 ± 0.052	25–35	0.782 ± 0.012	0.442 ± 0.052	0.127
36:64 CO:air	0.073 ± 0.006	34–48	0.104 ± 0.016	34–48	0.748 ± 0.006	0.256 ± 0.042	0.095
75:25 CO:air	0.138 ± 0.009	34–48	0.351 ± 0.038	34–48	0.807 ± 0.022	0.136 ± 0.083	0.064
4 h pre-culture	0.129 ± 0.018	35–49	0.303 ± 0.046	35–49	0.796 ± 0.029	0.491 ± 0.015	0.372
24 h pre-culture	0.115 ± 0.025	34–48	0.27 ± 0.098	34–48	0.779 ± 0.013	0.316 ± 0.027	0.132
10% inoculum	0.101 ± 0.022	25–35	0.137 ± 0.025	25–35	0.806 ± 0.025	0.425 ± 0.063	0.052
20% inoculum	0.068 ± 0.023	59–73	0.108 ± 0.029	59–73	0.741 ± 0.028	0.151 ± 0.131	0.013
Combined: 55 °C, pH 7.0, 0.08 mM Fe^2+^, no Ni^2+^, 75% CO; 4 h pre-culture; 10% inoculum	0.182 ± 0.009	34–58	0.566 ± 0.024	34–58	0.808 ± 0.01	0.323 ± 0.02	0.32

^a^Calculated between 24 h and 72 h cultivation time

### Effect of inoculum preparation on H_2_ productivity

The effect of different inoculum preparations on H_2_ production by *P. thermoglucosidasius* DSM 6285 was determined using different inoculum sizes (2%, 10%, 20% v/v) and incubation times of the 2nd pre-cultures (4 h, 12 h, 24 h). Maximum OD_600_ was observed after ~ 72 and 24 h when inocula (incubated for 12 h) of 2% (OD_600_ = 0.620 ± 0.137) and 10% (OD_600_ = 0.923 ± 0.054) were added, respectively (Fig. 1, Additional file 2). The highest OD_600_ was observed when the highest cell concentration (20%) was added, with a maximum absorbance of 1.057 ± 0.063 ~ 7 h post-inoculation (Additional file 2). However, during the aerobic growth phase the highest growth rate was observed for the 2% inoculum (0.14 1/h) (Table 2). Oxygen reached its minimal plateau ~ 24 h post-inoculation for all three inoculum sizes, and H_2_ was first detected at this time when a 10% inoculum (0.021 ± 0.010 mmol) and 20% inoculum (0.024 ± 0.015 mmol) was used (Fig. 2b). By contrast, with the 2% inoculum, 0.009 ± 0.003 mmol of H_2_ could already be detected ~ 12 h after inoculation and 0.103 ± 0.027 mmol was detected after 24 h. CO was mostly depleted after ~ 83 h (10% inoculum, 2% inoculum), while 0.179 ± 0.239 mmol CO was still present at this time point with the 20% inoculum (Fig. 2b). When considering hydrogen production rate, the highest production rate was observed with the 10% inoculum (0.101 ± 0.022 mmol/h), and occurred between 25 and 35 h post-inoculation (Table 2). A slightly lower maximum production rate (0.094 ± 0.016 mmol/h) was seen with the 2% inoculum and occurred later (between 35–48 h post-inoculation) than with the 10% inoculum. Maximum production rate for the highest inoculum size (20%) was achieved only between the 59–73 h time intervals and was 48.52% and 38.24% less than was observed with the 10% and 2% inocula, respectively (Table 2). Though the specific production rate was the highest for the 2% inoculum (0.14 mmol/h/OD_600_), the overall H_2_ yield is highest for the 10% inoculum (Table 2), and this inoculum size was thus selected as the optimal parameter for further experiments.

Substantial differences in the growth, maximum production rates and H_2_ yields could also be observed when distinct pre-culture inocula ages were evaluated. For the 4 h pre-culture, it took ~ 12 h to reach its maximum absorbance (OD_600_ = 0.50 ± 0.01), while it took ~ 24 h for the 12 h (0.620 ± 0.137) and 24 h inocula (0.56 ± 0.124) to reach their maximum absorbances (Fig. 1, Additional file 3). Growth rates during the aerobic phase also differed. Cultures inoculated with a 2nd pre-culture cultivated for 4 h, showed the highest growth rate (0.327 1/h) (Table 2). While in all cases maximum production rate occurred between the same time points, 36–48 h post-inoculation, the maximal production rate and H_2_ yield were highest with the 4 h pre-inoculum (0.129 ± 0.018 mmol/h between 35 and 49 h; 0.796 ± 0.029 mmol H_2_/mmol CO). The same pattern was observed for the specific production rate (Fig. 2c, Table 2).

### Effect of pH and temperature on hydrogen production

Different medium pHs (5.5, 7.0, 8.5) and cultivation temperatures (50 °C, 55 °C, 60 °C) were evaluated for their effects on hydrogen production. The maximum OD_600_ was observed in cultures maintained at 55 °C (maximum OD_600_ = 0.854 ± 0.141 after 48 h; OD_600_ = 0.846 ± 0.118 after 24 h), followed by growth at 50 °C (maximum OD_600_ = 0.787 ± 0.039 after 24 h) and 60 °C (maximum OD_600_ = 0.620 ± 0.137 after 24 h) (Fig. 1, Additional file 4). During aerobic growth, the growth rate during the cultivation at 55 °C was highest (0.172 1/h), followed by 50 °C (0.163 1/h) and 60 °C (0.140 1/h) (Table 2). Depletion of O_2_ (~ 24 h) and CO (after ~ 72 h) occurred earlier at 55 °C and 60 °C than at 50 °C (O_2_ depletion after ~ 36 h; 1.346 ± 0.772 mmol CO after 72 h) (Fig. 2d, Additional file 4). This correlated with both the higher maximum H_2_ production rates and yields observed at the higher temperatures. Highest production rates at these temperatures occurred between 34 and 48 h post-inoculation, while at 50 °C this was only achieved in the last part (73–82 h) of the experiment (Fig. 2d). Only marginal differences in both maximum production rates and yield were observed with the other experimental temperatures, with both factors being slightly higher (0.004 mmol/h more H_2_ produced between 34 and 48 h; yield: 0.085 mmol more H_2_ per mmol CO) at 55 °C than at 60 °C (Table 2). Given these marginal differences and the superior growth rate at 55 °C, this temperature was selected as the optimal condition for further experimentation although the specific production rate was the lowest during the cultivation at 55 °C.

More substantial differences could be observed for *P. thermoglucosidasius* grown in media adjusted prior inoculation to pH 5.5, 7.0 and 8.5. The highest OD_600_ was observed for the pH 7.0 cultures (maximum absorbance of 0.620 ± 0.137 after ~ 24 h), while *P. thermoglucosidasius* grew least well at pH 8.5 (maximum absorbance of 0.463 ± 0.018 after 6 h) (Additional file 5). The growth rate (aerobic phase) during the cultivation with a pH of 7.0 was also higher than with the other two medium pHs. Differences in oxygen consumption were also observed. Whereas O_2_ reached its minimal plateau after ~ 24 h for the cultivations with medium pH 7.0 and 8.5, it only reached its minimum after 48 h at pH 5.5 (Fig. 1, Additional file 5). The highest maximal H_2_ production rate (0.122 ± 0.005 mmol/h) and yield (0.786 ± 0.018 mmol H_2_/mmol CO) were observed at pH 8.5. However, maximum productivity occurred substantially later (59–72 h post-inoculation) when a medium pH of 7.0 was used (35–48 h post-inoculation) (Fig. 2e, Additional file 5). By contrast, the specific production rate was higher at pH 5.5 and pH 8.5 than at pH 7.0, but occurred 12–24 h later. As the concept of parametric optimization should not be considered solely on the basis of yield, but also the time-efficiency of the process, the pH of 7.0 was selected as the optimum condition for H_2_ production.

### Effect of nickel and iron concentration on H_2_ production

Both the carbon monoxide dehydrogenase (CODH) and the hydrogenase that catalyses the WGS contain nickel (Ni^2+^) and iron (Fe^2+^) as co-factors (Can et al. 2014; Peters et al. 2015; Mohr et al. 2018a). Exogenous nickel and iron were added to the medium in order to evaluate their effect on hydrogenogenesis. The addition of nickel resulted in a maximal absorbance (OD_600_) of 0.486 ± 0.022 after 24 h. When more iron was added, the OD_600_ rose to a maximum of 0.572 ± 0.066 after 12 h (Additional file 6). By contrast, the control fermentation (no additional nickel or iron) showed a higher maximum absorbance of 0.620 ± 0.137 after 24 h (Additional file 6). However, the aerobic growth rate was less if no extra nickel or more iron was added. In all set-ups oxygen attained its minimum after ~ 24 h. Hydrogen was detected for the first time after ~ 12 h without added iron (0.009 ± 0.003 mmol) and after 24 h with additional iron (0.199 ± 0.038 mmol), while when nickel was added H_2_ production only commenced after 24 h (0.017 ± 0.012 mmol). CO was completely consumed after 82 h when iron was added and in the control samples, whereas 0.144 ± 0.069 mmol was still available when nickel was added (Fig. 2f). The addition of nickel also resulted in a substantially lower maximal production rate (0.078 mmol/h between 48 and 60 h post-inoculation), which was 17.02% less than was achieved without addition of nickel and iron, 36–48 h post-inoculation. By contrast, addition of iron resulted in a higher maximum production rate (0.115 mmol/h), which occurred 12 h earlier than the without the addition of iron. Furthermore, the overall yield was ~ 2% and 8% higher with the addition of iron than when nickel was added or when no nickel or added iron were included (Table 2). Therefore, the addition of 0.08 mM FeSO_4_·7H_2_O was selected for subsequent experiments.

### Optimized hydrogen production

**Fig. 3:**
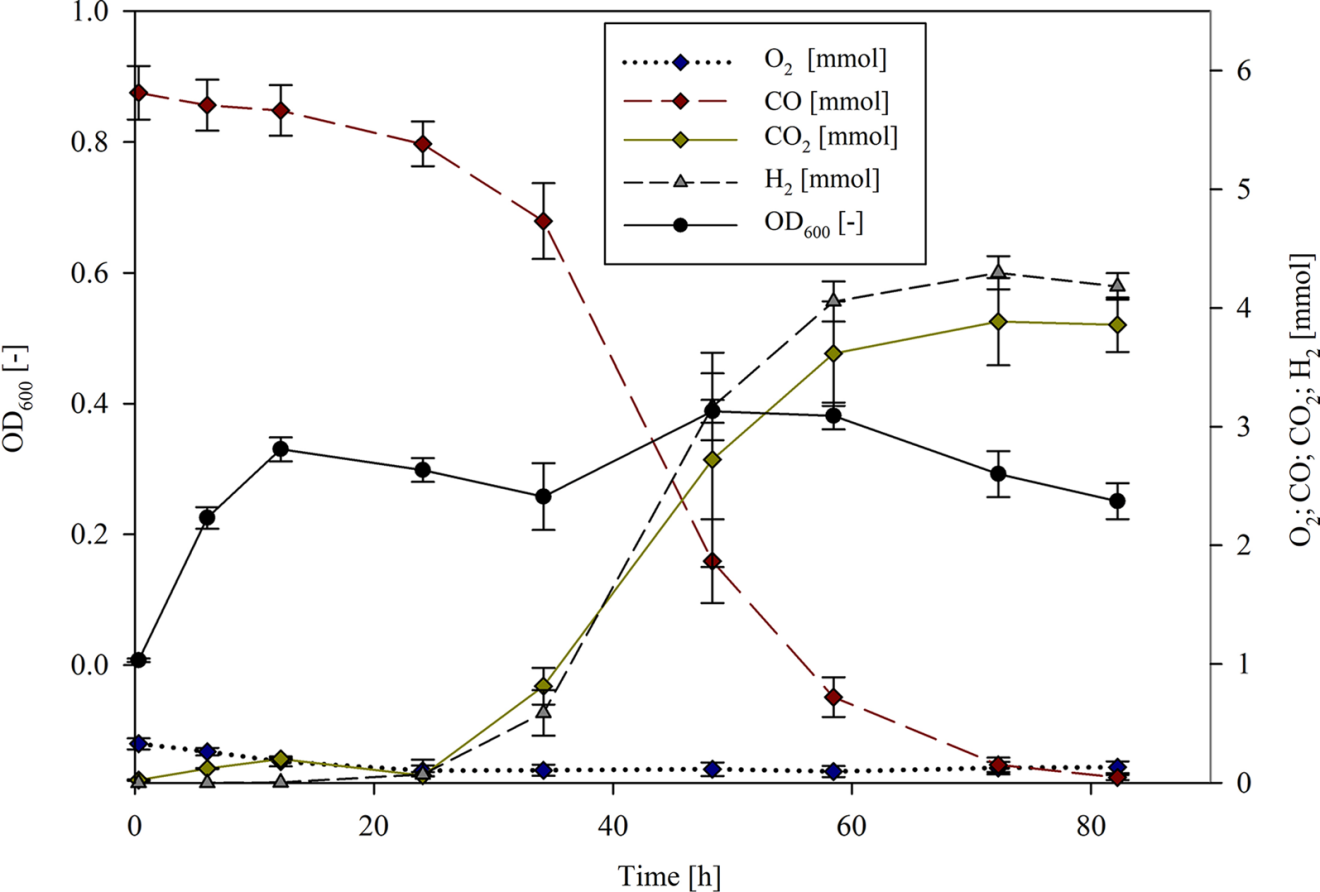
Growth curve and gas composition during the cultivation of *P.* *thermoglucosidasius* DSM 6285 with the combined superior parameters (55 °C, pH 7.0, addition of 0.08 mM FeSO_4_·7H_2_O, 75:25 initial gas atmosphere CO:air ratios, 4 h incubation time of the 2nd pre-culture, 10% inoculum size)

From the experiments evaluating the individual parameters, all tested parameters were observed to have substantial effects on both hydrogen yield and maximum production rates. In a further experiment, the effects of a combination of the optimum parameters (55 °C, pH 7.0, addition of FeSO_4_·7H_2_O (0.08 mM), 75:25 CO:air ratios (initial gas atmosphere), 4 h incubation time of the 2nd pre-culture, 10% inoculum size) on hydrogenogenesis was established. In this experiment, *P. thermoglucosidasius* growth to a maximum absorbance was observed after ~ 48 h (OD_600_ = 0.388 ± 0.018), while O_2_ was depleted earlier (after ~ 24 h). At this time, H_2_ was detected for the first time (0.071 ± 0.02 mmol) (Fig. 3).

Comparison to the previously evaluated control set-up (60 °C, pH 7.0, 0.04 mM FeSO_4_·7H_2_O, 50:50 CO:air ratio, 12 h incubation time of the 2nd pre-culture, 2% inoculum size; Mohr et al. 2018b) showed a modest increase in H_2_ yield (2% higher) when the optimized conditions were used (Table 2). However, marked increases in both the maximum (1.9× higher) and specific H_2_ production rate (3.5× higher) could be observed with the optimized parameters, occurring ~ 35–48 h post-inoculation in both cases. These factors were also substantially higher than each of the single tested parameters, with a 1.6× and 5.4× fold increase in specific H_2_ production rate for the best (75:25 CO:air ratio) and worst (36:64 CO:air ratio) performing individual parameter, respectively (Table 2).

## Discussion

A critical aspect of microbial fermentations that involve gas as the main substrate or e^−^ acceptor is the solubility of the gas and the threshold concentration that does not inhibit the metabolism of the microorganisms (Bertsch and Müller 2015). In general, high gas concentrations can have an inhibitory effect while low gas concentrations can result in a low volumetric mass transfer coefficient resulting in limited substrate availability (Daniell et al. 2012; Mohammadi et al. 2014). This was evident in the fermentations with *P. thermoglucosidasius* DSM 6285 as less growth (biomass) was observed with increasing CO concentrations and concomitantly lower concentration of oxygen as terminal electron acceptor during the aerobic growth phase. However, poorer growth at higher CO concentrations did not have a negative effect on the hydrogenogenic capacity of *P. thermoglucosidasius* DSM 6285, with the highest H_2_ production rate observed with the 75:25% CO:air mixture. The higher production rate with 75% CO, which grew to the lowest optical density, suggests that hydrogen productivity is a function of the availability of CO, rather than being dependent on the amount of biomass. To investigate the influence of the amount of biomass prior the hydrogen production phase, cultivations in bottles were undertaken using different inoculum sizes.

The size and age of inocula can have substantial effects on hydrogen fermentations, as has been observed in the fermentative thermophile *Thermoanaerobacterium thermosaccharolyticum* and the photosynthetic purple non-sulphur bacterium *Rhodobacter sphaeroides* (Japaar et al. 2011; Seengenyoung et al. 2011). The highest production rate was detected with the 10% inoculum size, while the lowest production rate was achieved with the highest inoculum size (20%). Similar results were obtained with the fermentative H_2_-producer *Bacillus coagulans* IIT-BT S1, where higher H_2_ production rates were observed with a 10% inoculum volume, but decreased with larger (15% and 20%) inoculum sizes (Kotay and Das 2007). As such, H_2_ production appears not to be directly linked to the amount of biomass but may rather be a function of the physiological state of *P. thermoglucosidasius*. To confirm this hypothesis, different cultivation times (4 h, 12 h, 24 h) of the 2nd pre-culture were tested. Although the maximum production rate was detected at the same time points, H_2_ production with the shortest incubation time of the 2nd pre-culture (4 h) showed the highest production rate. The 4 h pre-cultures may be in the lag growth phase preceding exponential growth (12–24 h), the preparative phase where bacteria adapt optimally to new environments (i.e., the exposure of *P. thermoglucosidasius* to CO) (Bertrand 2019). This pre-adaptive physiological state may explain the highest production rate observed with the 4 h pre-culture. Similarly, the lower H_2_ production rates with the 20% inoculum size may be due to the cells reaching the post-lag exponential phase more rapidly than the optimal 10% inoculum size.

*Parageobacillus thermoglucosidasius* strains grow optimally at temperatures of 61–63 °C and an initial medium pH of 6.5–8.5 (Suzuki et al. 1984). The strain utilized in this study, DSM 6285, is reported to grow optimally at 55 °C, with some growth at 75 °C (Gurujeyalakshmi and Oriel 1989). In the current study, a growth temperature of 55 °C and a medium pH of 7.0 resulted in optimal H_2_ production. Although the highest H_2_ production rate was obtained with the pH = 8.5 set up, the lag phase between oxygen consumption and the commencement of hydrogen production was substantially longer (24 h later than at pH 7.0).

Nickel (Ni^2+^) and iron (Fe^2+^) are both essential co-factors in the catalytic sites of a broad range of enzymes (Waldron and Robinson 2009), and both the Ni–Fe CODH and Ni–Fe group 4a hydrogenase that catalyse the WGS are reported to contain both of these co-factors (Mohr et al. 2018a). Thus, the addition of both of these elements to the *P.* *thermoglucosidasius* growth medium might be expected to have a positive effect on hydrogenogenesis. When doubling the amount of Fe^2+^ (0.08 mM FeSO_4_·7H_2_O) normally added to mLB medium, there was an evident decrease in the lag phase between oxygen consumption and hydrogen production and the maximum H_2_ production rate was 8% higher than at lower concentrations. However, the addition of NiSO_4_·6H_2_O had a negative impact on both the growth of *P. thermoglucosidasius* DSM 6285, the length of the pre-hydrogenogenic lag phase, H_2_ yield and maximum H_2_ production rate. A study of the effects of nickel on H_2_ production by anaerobic sludge bacteria showed that increasing the nickel concentration from 0.0 mM up to 0.01 mM led to an increase of hydrogen production, while higher nickel concentration had a negative effect on hydrogen production (Wang and Wan 2008). Furthermore, the lag phase of hydrogen production could be decreased to 6 h by using 0.01 mM nickel (Wang and Wan 2008). As such, further fine-tuning of the amount of nickel added may be necessary for improved *P. thermoglucosidasius* hydrogenogenesis.

The current study highlights that WGS catalyzed hydrogenogenesis in *P. thermoglucosidasius* is a finely balanced process with variations in all the tested operational parameters having either a positive or negative impact on H_2_ yield, maximal (specific) production rates, as well as the time frame of the lag phase preceding hydrogenogenesis and the growth. The optima for each parameter combined in a further experiment resulted in higher production rate compared to set ups in which individual parameters were tested separately. This study can serve as a basis for up-scale fermentations. However, the effects of additional parameters such as the stirrer rate and flow rate of the feed gas inherent to up-scale fermentations will also need to be evaluated.

Hydrogenogenesis via the WGS in *P. thermoglucosidasius* is a finely balanced process, which is influenced by key operational parameters. While some parameters such as temperature and initial medium pH reflect the optimum growth conditions for *P. thermoglucosidasius* others such as the age of the pre-culture and inoculum volume are more complex and may rather indicate the importance of the physiological state of *P. thermoglucosidasius* on its hydrogenogenic capacity. Further investigations, including gene expression analysis and metabolic profiling may shed light on additional factors influencing hydrogen production which, together with additional fine-tuning of operational parameters, can be used to develop up-scale fermentations with a continuous CO feed for commercial hydrogen production using the facultatively anaerobic thermophilic carboxydotroph *P. thermoglucosidasius*.

## Supplementary information

**Additional file 1.** Effect of initial gas composition on H_2_ production. OD_600_ and gas composition during the cultivation of *P. thermoglucosidasius* DSM 6285 with an initial gas atmosphere of (A) 36% CO + 64% air (B) 50% CO + 50% air (C) 75% CO + 25% air.

**Additional file 2.** Effect of inoculum preparation on H_2_ production—inoculum size. OD_600_ and gas composition during the cultivation of *P. thermoglucosidasius* DSM 6285 with different inoculum sizes of (A) 2% (B) 10% and (C) 20%.

**Additional file 3.** Effect of inoculum preparation on H_2_ production—incubation time of the 2nd pre-culture. OD_600_ and gas composition during the cultivation of *P. thermoglucosidasius* DSM 6285 with variations in the incubation time of the 2nd pre-culture: (A) 4 h (B) 12 h (C) 24 h.

**Additional file 4.** Effect of cultivation temperature on H_2_ production. OD_600_ and gas composition during the cultivation of *P. thermoglucosidasius* DSM 6285 with different cultivation temperatures: (A) 50 °C (B) 55 °C (C) 60 °C.

**Additional file 5.** Effect of initial pH on H_2_ production. OD_600_ and gas composition during the cultivation of *P. thermoglucosidasius* DSM 6285 with different pH set ups: (A) pH 5.5 (B) pH 7.0 (C) pH 8.5.

**Additional file 6.** Effect of Nickel and Iron concentration on H_2_ production. OD_600_ and gas composition during the cultivation of *P. thermoglucosidasius* DSM 6285 with addition of trace elements: (A) 0.3 mM NiSO_4_·6H_2_O + 0.04 mM FeSO_4_·7H_2_O and (B) 0.080 mM FeSO4·7H_2_O (C) 0.04 mM FeSO_4_·7H_2_O.

## Data Availability

The data supporting the conclusions of this article are included in the article. Data and materials can also be requested from the corresponding author.
