# Supplementary figures and images for: Effects of different operating parameters on hydrogen production by Parageobacillus thermoglucosidasius DSM 6285
Source: AMB Express. 2019 Dec 23;9:207. doi: 10.1186/s13568-019-0931-1 (PMC6928187; doi:10.1186/s13568-019-0931-1)

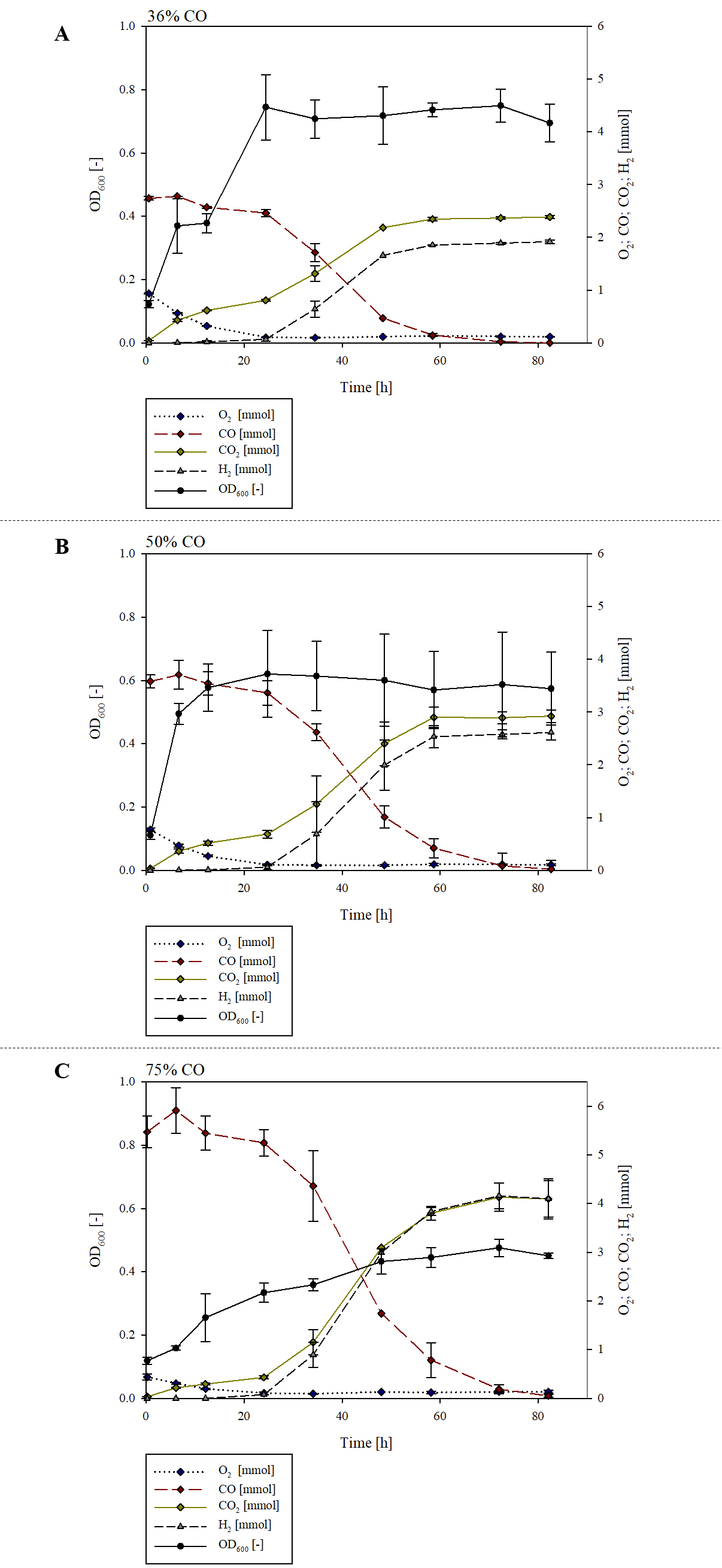

Supplement: Supplementary file 1 — Additional file 1. Effect of initial gas composition on H2 production. OD600 and gas composition during the cultivation of P. thermoglucosidasius DSM 6285 with an initial gas atmosphere of (A) 36% CO + 64% air (B) 50% CO + 50% air (C) 75% CO + 25% air. [file 13568_2019_931_MOESM1_ESM.png]

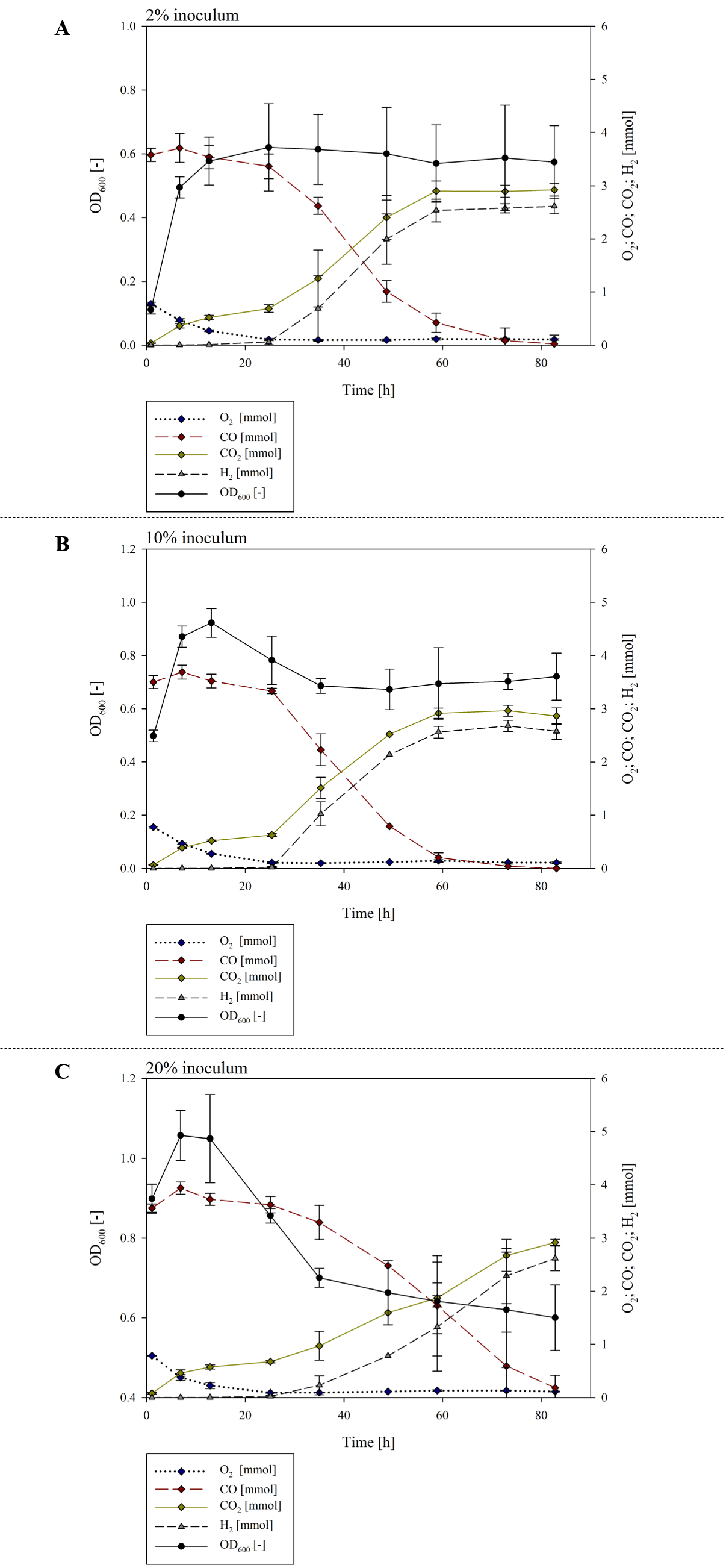

Supplement: Supplementary file 2 — Additional file 2. Effect of inoculum preparation on H2 production—inoculum size. OD600 and gas composition during the cultivation of P. thermoglucosidasius DSM 6285 with different inoculum sizes of (A) 2% (B) 10% and (C) 20%. [file 13568_2019_931_MOESM2_ESM.png]

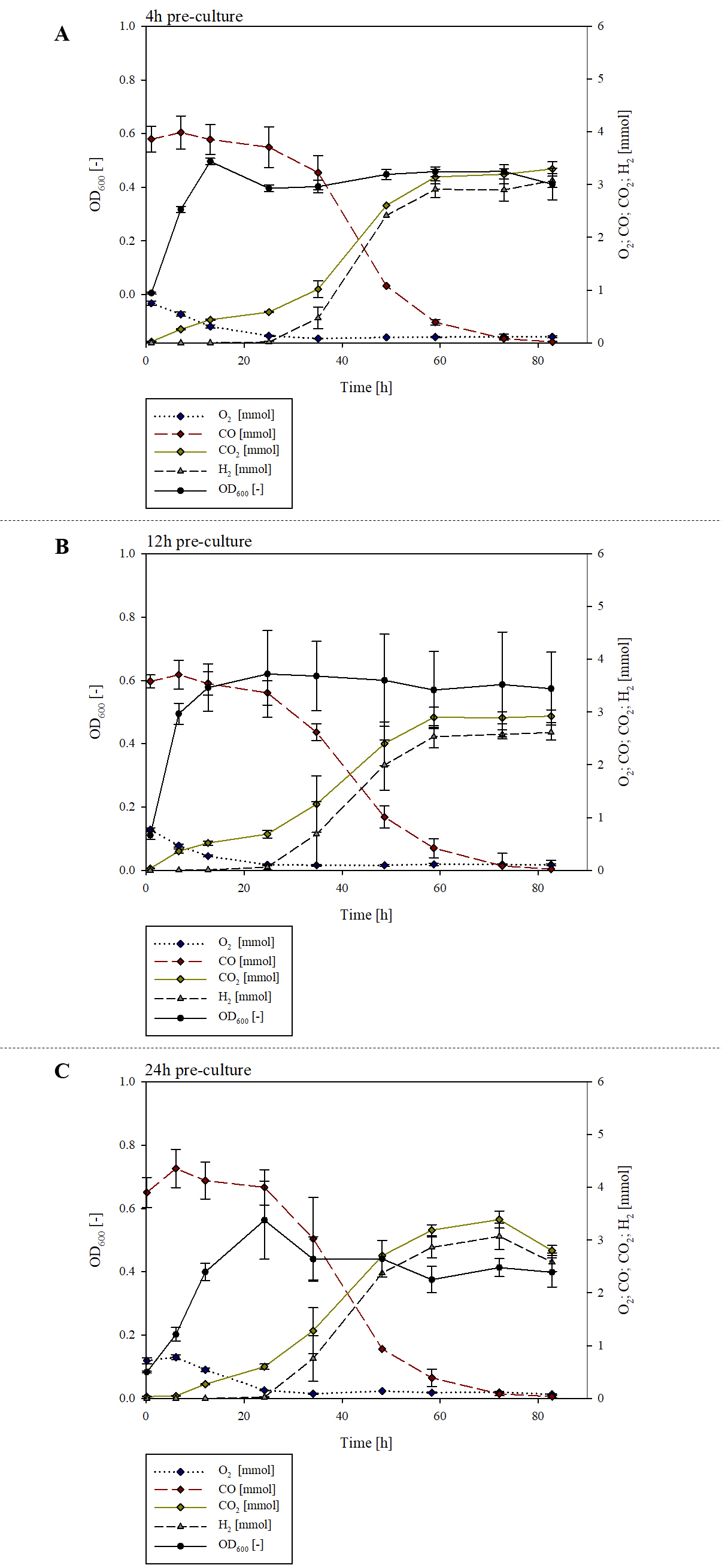

Supplement: Supplementary file 3 — Additional file 3. Effect of inoculum preparation on H2 production—incubation time of the 2nd pre-culture. OD600 and gas composition during the cultivation of P. thermoglucosidasius DSM 6285 with variations in the incubation time of the 2nd pre-culture: (A) 4 h (B) 12 h (C) 24 h. [file 13568_2019_931_MOESM3_ESM.png]

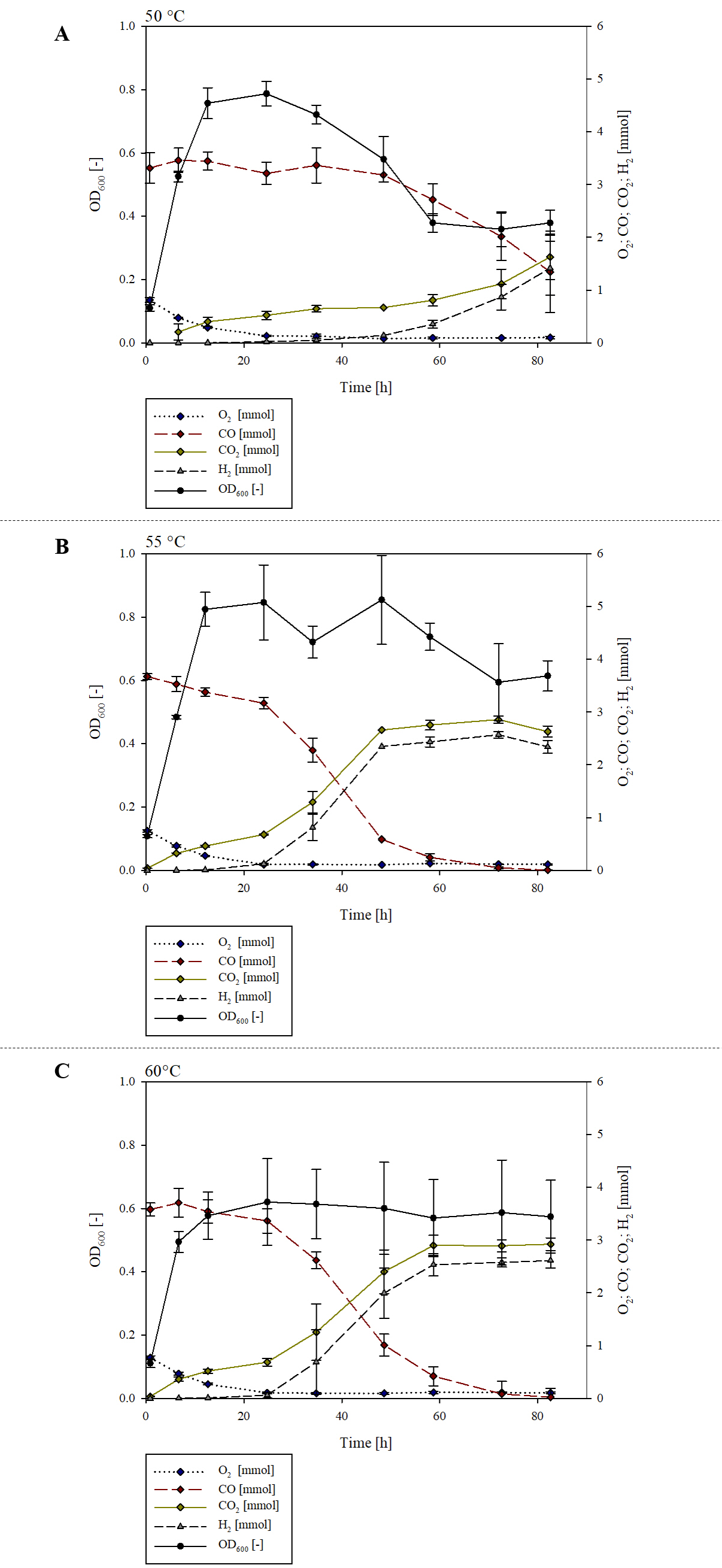

Supplement: Supplementary file 4 — Additional file 4. Effect of cultivation temperature on H2 production. OD600 and gas composition during the cultivation of P. thermoglucosidasius DSM 6285 with different cultivation temperatures: (A) 50 °C (B) 55 °C (C) 60 °C. [file 13568_2019_931_MOESM4_ESM.png]

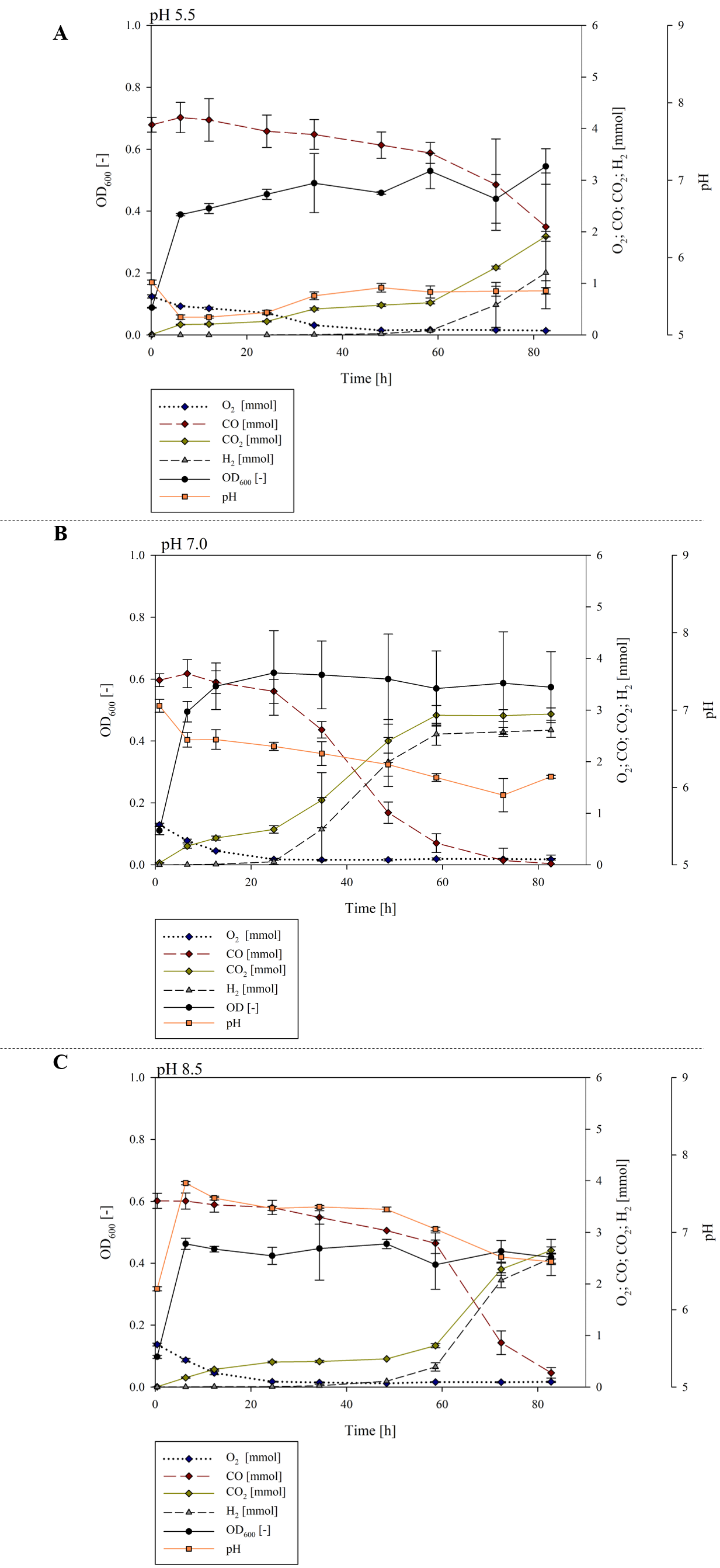

Supplement: Supplementary file 5 — Additional file 5. Effect of initial pH on H2 production. OD600 and gas composition during the cultivation of P. thermoglucosidasius DSM 6285 with different pH set ups: (A) pH 5.5 (B) pH 7.0 (C) pH 8.5. [file 13568_2019_931_MOESM5_ESM.png]

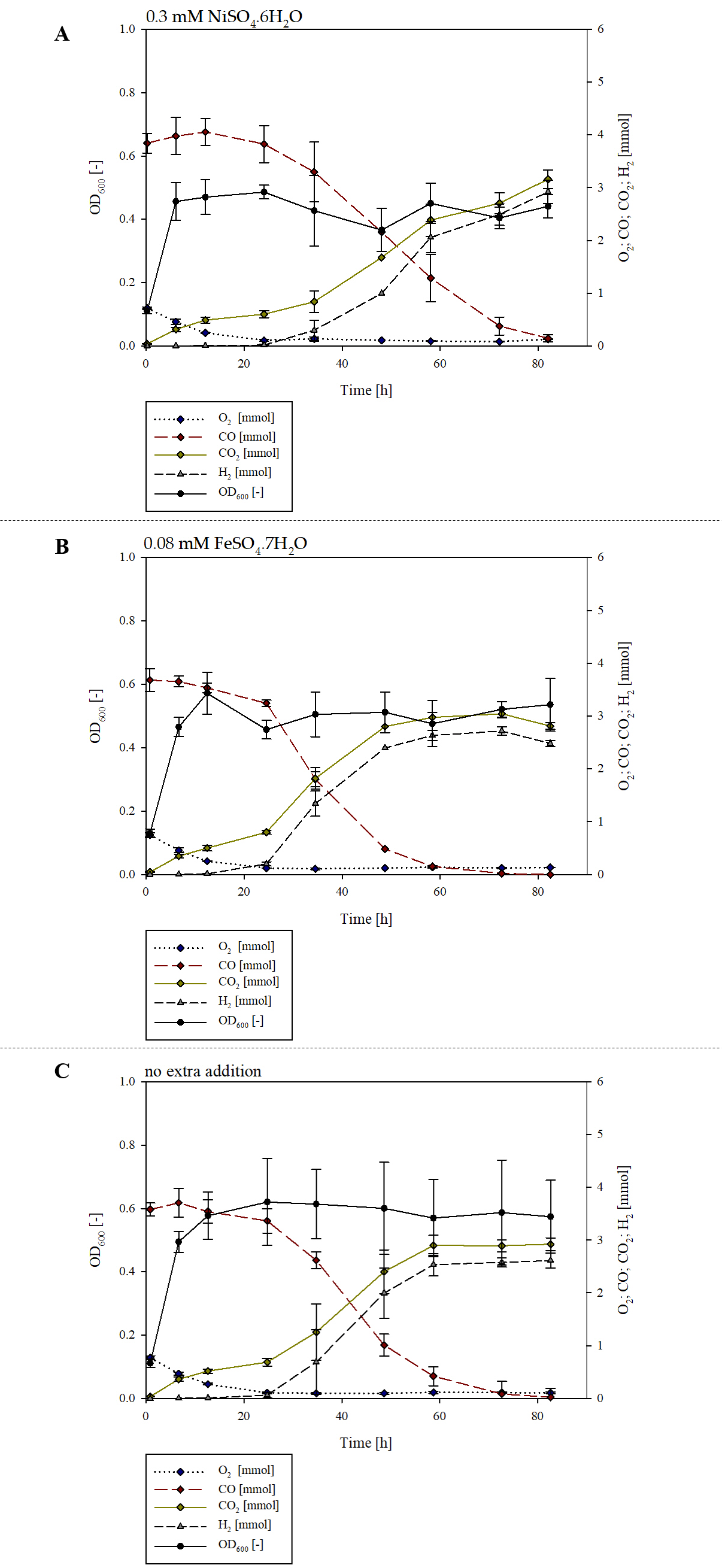

Supplement: Supplementary file 6 — Additional file 6. Effect of Nickel and Iron concentration on H2 production. OD600 and gas composition during the cultivation of P. thermoglucosidasius DSM 6285 with addition of trace elements: (A) 0.3 mM NiSO4·6H2O + 0.04 mM FeSO4·7H2O and (B) 0.080 mM FeSO4·7H2O (C) 0.04 mM FeSO4·7H2O. [file 13568_2019_931_MOESM6_ESM.png]
